# I want to media multitask and I want to do it now: Individual differences in media multitasking predict delay of gratification and system-1 thinking

**DOI:** 10.1186/s41235-016-0048-x

**Published:** 2017-01-30

**Authors:** Dan Schutten, Kirk A. Stokes, Karen M. Arnell

**Affiliations:** Department of Psychology, Brock University, 1812 Sir Isaac Brock Way, St. Catharines, ON L2S 3A1 Canada

**Keywords:** Media multitasking, MMI, Decision making, Delay discounting, Delay of gratification, Cognitive reflection test, CRT, Impulsivity

## Abstract

Media multitasking, the concurrent use of multiple media forms, has been shown to be related to greater self-reported impulsivity and less self-control. These measures are both hallmarks of the need for immediate gratification which has been associated with fast, intuitive ‘system-1’ decision making, as opposed to more deliberate and effortful ‘system-2’ decision making. In Study 1, we used the Cognitive Reflection Task (CRT) to examine whether individuals who engage heavily in media multitasking differ from those who are light media multitaskers in their degree of system-1 versus system-2 thinking. In Study 2 we examined whether heavy and light media multitaskers differ in delay of gratification, using the delay discounting measure which estimates the preference for smaller immediate rewards, relative to larger delayed rewards in a hypothetical monetary choice task. We found that heavy media multitaskers were more likely than light media multitaskers to endorse intuitive, but wrong, decisions on the CRT indicating a greater reliance on ‘system-1’ thinking. Heavy media multitaskers were also willing to settle for less money immediately relative to light media multitaskers who were more willing to wait for the larger delayed reward. These results suggest that heavy media multitaskers have a reactive decision-making style that promotes current desires (money, ease of processing) at the expense of accuracy and future rewards. These findings highlight the potential for heavy media multitaskers to be at risk for problematic behaviors associated with delay discounting – behaviors such as substance abuse, overeating, problematic gambling, and poor financial management.

## Significance

As cognitive psychologists we often measure the ability to pay attention to multiple stimuli in the laboratory using rather artificial computer tasks. However, as personal media devices have become more ubiquitous, dividing our attention amongst several media has become a daily “real-world” behavior. Media multitasking refers to the concurrent use of multiple media forms. Casual observation of the behavior of others in our daily life suggests that individual’s differ in their tendency to media multitask. Individual differences in everyday media multitasking have typically been estimated using the Media Multitasking Index (MMI) of Ophir et al. (2009), and MMI scores have been shown to predict various aspects of cognitive processing, personality, and affect. Here, we provide evidence that greater media multitasking is related to decision-making style in that it predicts greater use of fast, intuitive ‘system-1’ decision making versus slower, more effortful ‘system-2’ decision making. We also show that heavy media multitaskers (HMMs) show a preference for smaller immediate rewards, relative to larger delayed rewards in a delay discounting task that measures delay of gratification. We propose that HMMs have a more reactive decision-making style that promotes current desires (money and ease of processing) at the expense of accuracy and future rewards. Greater delay discounting has previously been associated with behaviors, such as substance abuse, overeating, problematic gambling, and poor financial management, suggesting that the decision-making style of HMMs may put them at risk for these problematic lifestyle choices.

## Background

As personal media devices have become more ubiquitous, dividing our attention amongst several media has become a daily real-world behavior. For example, it is common practice for many students to watch television and/or listen to music, while reading their textbook, while texting friends, while checking social media updates. The term media multitasking refers to examples such as this where there is concurrent use of multiple media forms to simultaneously accomplish different goals (Ophir et al., 2009). Why do some individuals choose to media multitask while others avoid it? Why do some people seem unable to put their phone down and focus on the primary task that they are trying to accomplish? Do individuals who simultaneously use multiple forms of media frequently differ from those who choose to focus on one form of media at a time?

Individual differences in media multitasking have typically been estimated using the Media Multitasking Index (MMI) of Ophir et al. (2009) where participants’ self-reports on how much each form of media is used with each other media form allow a calculation of the frequency of media multitasking for that individual. MMI scores allow one to examine the cognitive and personality characteristics that are associated with media multitasking and identify those characteristics that can discriminate HMMs from light media multitaskers (LMMs). A profile is emerging that suggests that HMMs process information with less goal-relevant selectivity in both visual search (Cain & Mitroff, 2011; Lui & Wong, 2012) and working memory tasks (Ophir et al., 2009; Sanbonmatsu, Strayer, Medeiros-Ward, & Watson, 2013). This difficulty in ignoring irrelevant information has been interpreted as HMMs having reduced cognitive control abilities – a supposition supported by the finding that HMMs have reduced gray-matter density in the anterior cingulate cortex (Loh & Kanai, 2014), an area implicated in the coordinated control of goal-directed behavior (e.g., Bush, Luu, & Posner, 2000). Frequent media multitasking does not appear to lead to improved multitasking performance or the ability to accurately evaluate one’s own multitasking ability. Sanbonmatsu et al. (2013) found that media multitasking scores were negatively related to actual multitasking ability on an executive control working memory task even though media multitasking scores were positively related to self-perceived multitasking ability.

Media multitasking has also been shown to be related to greater self-reported impulsivity (Minear, Brasher, McCurdy, Lewis, & Younggren, 2013; Sanbonmatsu et al., 2013), greater sensation seeking (Duff, Yoon, Wang, & Anghelcev, 2014; Sanbonmatsu et al., 2013), more mind-wandering and everyday lapses of attention, (Ralph, Thomson, Cheyne, & Smilek, 2014), and lower self-reported self-control (Minear et al., 2013). Furthermore, Minear et al. (2013) observed that HMMs scored lower than LMMs on items from the Raven’s Advanced Progressive Matrices, but were also faster to give up on difficult items, perhaps reflecting their greater impulsivity, but also possibly a correct assessment that they could not solve the question, or an incorrect assessment that they had correctly solved the question.

## System-1 and system-2 thinking

The above results suggest that HMMs may engage in less deliberate and effortful thinking than LMMs. Kahneman and colleagues (e.g., Kahneman, 2011; Kahneman & Shane, 2005; Morewedge & Kahneman, 2010) distinguish between system-1 and system-2 thinking during decision making (see also Gilbert, 1989; Stanovich & West, 2002 for similar dual-process models). System-1 thinking is fast and intuitive, while system-2 thinking is slower, more effortful, and deliberate. While the answer “4” may readily pop into your head when you are given “2 × 2,” getting 306 when given “17 × 18” almost surely requires deliberate cognitive effort using system 2. System-1 thinking can be efficient and correct as in “2 × 2,” but it also underlies the common, but erroneous, performance seen in common decision-making heuristics such as availability and representativeness (e.g., Toplak, West, & Stanovich, 2011).

In Study 1 we specifically look for possible differences between light versus HMMs’ thinking style using Frederick’s Cognitive Reflection Test (CRT, Frederick, 2005). The CRT consists of three questions where the correct answer, that one could readily achieve using system-2 thinking, differs from the intuitive, immediate answer delivered by system-1 thinking. For example, one of the items reads “If it takes five machines 5 minutes to make five widgets, how long would it take 100 machines to make 100 widgets?” If an individual applied little to no cognitive effort and relied on the automatic system-1 answer, then they would likely arrive at the intuitive, but wrong, answer of 100 min. Instead, if an individual thought through the question deliberately most would readily realize that every machine is making one widget every 5 min, so it would still take 5 min for 100 machines to make 100 widgets. Therefore, the total number of intuitive answers provides a measure of reliance on system-1 thinking, and the total number of correct answers provides a measure of reliance on system-2 thinking.^1^ Low system-2 scores on the CRT have been shown to predict increased use of decision-making heuristics and shortcuts (Toplak et al., 2011) and reduced willingness to delay gratification (Frederick, 2005). The CRT has also been referred to as a “potent measure of the tendency toward miserly processing” (Toplak et al., 2011, p. 1275). The CRT score is generally treated as trait-variable, reflecting an individual’s disposition and/or ability (see Campitelli & Gerrans, 2014 for a discussion). This is supported by studies showing that CRT scores relate to traits and dispositions, such as gender (Frederick, 2005; Campitelli & Gerrans, 2014; Pennycook, Cheyne, Koehler, & Fugelsang, 2016), scores on the Need for Cognition Scale (Frederick, 2005; Pennycook et al., 2016), and personality facets and real-world decision-making styles (Juanchich, Dewberry, Sirota, & Narendran, 2016), as well as ability measures such as Scholastic Assessment Test (SAT) scores (Frederick, 2005), working memory scores, and performance on various subscales of the Wechsler Abbreviated Scale of Intelligence (Toplak et al., 2011). Based on previous findings of greater self-reported impulsivity and reduced cognitive control for HMMs, we predict that HMMs will have fewer correct answers, and more intuitive answers, on the CRT than LMMs, reflective of a more intuitive, less effortful, system-1 processing style.

## Delay of gratification

Lower system-2 scores on the CRT predict reduced delay of gratification (Frederick, 2005). Delay of gratification is the ability to forego immediate gratification in order to receive a larger reward at a later time (Mischel, Shoda, & Rodriguez, 1989). Delay of gratification is a form of self-regulation that has been shown to have important life outcomes. For example, preschool children who were able to delay gratification and resist eating one marshmallow immediately in order to receive two marshmallows several minutes later were rated 10 years later by their parents as having greater academic and social competence, a greater ability to plan, think ahead, and listen attentively, as well as being better at managing frustration and stress (Mischel, Shoda, & Peake, 1988).

One common way that delay of gratification is measured in adults is through a paradigm called delay discounting (e.g., Odum, 2011). Delay discounting refers to the extent to which an individual’s perceived value of a reward declines as the delay until its receipt increases (Mazur, 1987). Participants are presented with choices between small, immediate rewards and larger, delayed rewards. Those who have higher discounting rates (i.e., who discount future rewards more and, therefore, have a higher preference for immediate gratification) are thought to be more impulsive and to have less self-control (e.g., MacKillop et al., 2011). Indeed, delay discounting is associated with many important behavioral life outcomes including problematic gambling (Alessi & Petry, 2003), obesity (Epstein, Salvy, Carr, Dearing, & Bickel, 2010; Zhang & Rashad, 2008), poor financial management (Bidewell, Griffin, & Hesketh, 2006), and dependence on substances such as opioids (Madden, Petry, Badger, & Bickel, 1997), alcohol (Vuchinich & Simpson, 1998), nicotine (Baker, Johnson, & Bickel, 2003), and marijuana (Johnson et al., 2010). Delay discounting can also predict future substance misuse (e.g., Audrain-McGovern et al., 2009) and future cessation/treatment success (e.g., Krishnan-Sarin et al., 2007; Tucker, Vuchinich, & Rippens, 2002).

In Study 2 we look for possible differences in delay of gratification in LMMs and HMMs. Because impulsivity and self-control are important factors in delay discounting (e.g., Frederick, 2005), and greater media multitasking use has been shown to predict greater impulsivity and reduced self-control (e.g., Minear et al., 2013; Sanbonmatsu et al., 2013), we hypothesize that HMMs would show a reduced ability to delay gratification relative to low media multitaskers.

## Study 1

### Methods

#### Participants

Participants were 501 undergraduate students (417 were female) ranging in age from 17 years to 43 years (*M* = 19.63 years) who received research participation credit. Participants were run in person individually or in small groups. Some participants completed additional computer tasks or questionnaires that are not the focus of the present investigation. Of these, 206 also participated in Study 2. All participants completed the CRT prior to the MMI. The stopping rule was to run as many participants as possible “before the end of the winter term,” with a minimum goal of 400 participants. Data was not analyzed until the full *N* of 501 was achieved.

MMI scores could not be calculated for 45 of the participants who failed to complete the MMI correctly or fully (typically leaving one side of the diagonal blank in the matrix) and these were removed from the dataset. Two of the remaining participants failed to complete the CRT. Of the remaining participants, 303 also completed the Barratt Impulsivity Scale (BIS-11; Patton et al., 1995) after the CRT and before the MMI.

#### Measures

##### Media Multitasking Index – MMI

The MMI (Ophir et al., 2009) was used to measure an individual’s level of trait multitasking. Participants first indicated how many hours a week they use 12 different forms of media (e.g., video games, email, television), and then for each of the 12 types of media they reported how often they use each of the other 11 forms of media while using that form primarily. The allowable responses included: 0 = never, 1 = a little of the time, 2 = some of the time, to 3 = most of the time. For each media type, these responses were first divided by 3, then added, and this value was then multiplied by the total number of hours per week using that medium. These products were then added together and divided by the total number of hours per week using all media to get an overall MMI score where a higher MMI score represents greater chronic media multitasking.

##### Cognitive Reflection Test – CRT

The CRT (Frederick, 2005) contains three logic questions that suggest an immediately intuitive, but wrong, answer. The correct answer can be found quite readily once deliberate logical thought is applied. Participants received all three questions on a single page titled “Decision Making Questionnaire.” They were not given any instructions about how to answer the questions or about how long to take, and were simply told to provide the correct answer to the question in the blank space provided beside each question. The CRT correct score was calculated as the number of items out of three that were answered correctly. The CRT intuitive score was calculated as the number of items out of three that were answered with the intuitive but wrong answer.

##### Barratt Impulsivity Scale – BIS-11

The BIS-11 (Patton et al., 1995) is a widely used self-report questionnaire designed to measure impulsivity. It contains 30 statements (e.g., “I do things without thinking”) and participants indicated the extent to which the statement generally applies to them using a 4-point Likert scale that varies from “rarely/never” to “almost always/always.” After recoding reversed items, scores were summed to get an overall BIS score and BIS subscale scores for the three impulsivity factors: Attention, Motor, and Nonplanning.

## Results

The *N*, mean (*M*), range, and standard deviation (*SD*) are provided in Table 1 for all measures. MMI and BIS scores showed good variability and were normally distributed. For the CRT, overall, participants gave few correct answers and many intuitive ones. However, each of the four possible scores (0 to 3) was observed by at least 27 participants for each measure so individual differences were still evident. The typical sex difference (Frederick, 2005; Campitelli & Gerrans, 2014; Pennycook et al., 2016) was observed for CRT scores, where male participants gave significantly more correct answers (*M* = 0.88) than female participants (*M* = 0.41), *t*(496) = 4.59, *p* < .001, *d* = .513, and fewer intuitive answers (*M* = 1.83) than female participants (*M* = 2.32), *t*(496) = 4.29, *p* < .001, *d* = .494.

**Table 1:** Descriptive data for Study-1 measures

Measure	*N*	*M*	*SD*	Minimum	Maximum
MMI score	456	4.69	1.87	.11	10.08
CRT correct	454	0.42	.87	0	3
CRT intuitive	454	2.24	.95	0	3
BIS (impulsivity)	303	58.02	11.25	33	90

*MMI* Media Multitasking Index, *CRT* Cognitive Reflection Test, *BIS* Barratt Impulsivity Scale

As hypothesized, greater MMI scores were associated with fewer items correct on the CRT, *r*(455) = −.17, *p* < .001, and greater use of more intuitive wrong answers, *r*(455) = .17, *p* < .001, even when controlling for number of hours of media use per week (both *p*s = .001), and when controlling for sex of participant (both *p*s < .001). Also, as hypothesized, higher MMI scores predicted greater impulsivity with overall BIS scores *r*(303) = .24, *p* < .001, and on each of the Attention, Motor, and Nonplanning factors of the BIS (all *p*s < .01). Furthermore, controlling for BIS scores did not remove the relationships between MMI scores and CRT correct (partial *r* = −.17, *p* = .003), or CRT intuitive (partial *r* = .13, *p* < .05), measures, which would be expected given the null relationship observed here between BIS scores and CRT measures, *r*(322) = −.04, *p* = .48 for CRT correct, and *r*(322) = −.02, *p* = .70 for CRT intuitive (see Table 2).

**Table 2:** Correlations amongst Study-1 measures

Measure	1	2	3
1. MMI score	-		
2. CRT correct	−.17	-	
3. CRT intuitive	.17	−.83	-
4. BIS (impulsivity)	.24	−.02	−.02

*MMI* Media Multitasking Index, *CRT* Cognitive Reflection Test, *BIS* Barratt Impulsivity Scale

**Fig. 1:**
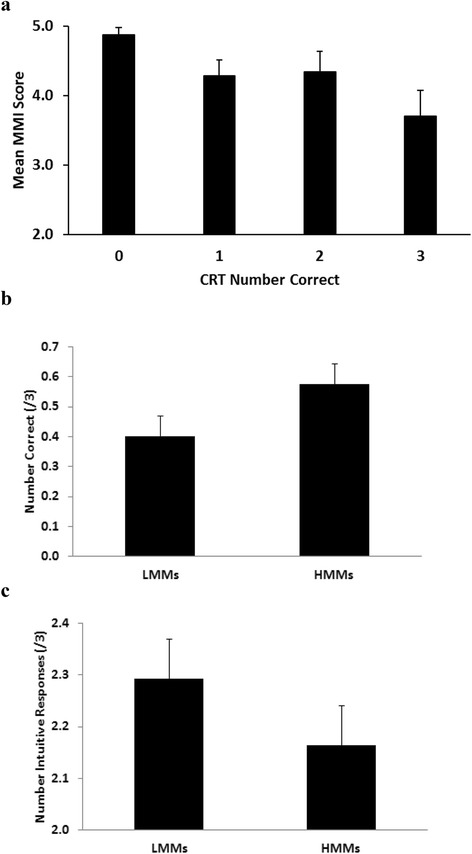
**a** Mean Media Multitasking Index (MMI) scores as a function of number correct on the Cognitive Reflection Test (CRT) in Study 1. **b** Mean number of correct responses on the CRT for low media multitaskers (LMMs) and heavy media multitaskers (HMMs) in Study 1. **c** Mean number of incorrect intuitive responses for LMMs and HMMs in Study 1. Error bars represent 1 standard error of the mean

Given that only four scores were possible on the CRT (0, 1, 2 or 3 correct), correct CRT score can also be used as an independent grouping variable with MMI score as the dependent variable. A between-participant one-way analysis of variance (ANOVA) showed a significant effect of CRT group, where MMI scores differed significantly across CRT groups, *F*(3,451) = 4.92, *p* = .002, partial *ƞ* ^*2*^ = .032, see Fig. 1a. Follow-up paired comparisons with the Bonferroni alpha correction showed a significantly higher MMI score for those with a correct CRT score of 0 than those with a correct CRT score of 3 (*p* < .05).

### Extreme groups’ comparisons

Individuals scoring more than 1 SD above the mean (i.e., MMI scores higher than 6.56) were classified as HMMs (*N* = 73) and individuals scoring more than 1 SD below the mean (i.e., MMI scores lower than 2.80) were classified as LMMs (*N* = 75); an approach that has been popular when using the MMI (e.g., Cain & Mitroff, 2011, Minear et al., 2013; Ophir et al., 2009).

Corroborating the correlational results, HMMs scored significantly lower overall on the CRT correct than did LMMs, *t*(145) = 2.73, *p* = .007, *d* = .457, and provided significantly more intuitive wrong answers than did LMMs, *t*(145) = 2.88, *p* = .005, *d* = .478 (see Fig. 1b, c). HMMs also showed significantly greater self-reported impulsivity on the BIS than LMMs, *t*(102) = 3.49, *p* < .001, *d* = .693. These results provide the first evidence that HMMs rely more on a fast and intuitive system-1 processing style.

## Study 2

### Methods

Participants were 206 Brock University undergraduate students (158 were female) ranging in age from 17 years to 43 years (*M* = 19.74 years) who participated voluntarily in this study in exchange for research credit. All participants were run in small groups and completed the delay discounting measure prior to the MMI. Instructions, completion, and scoring of the MMI were the same as in Study 1. The stopping rule was to run 200 participants, and experimental timeslots were posted to approximate this number with a few extra for safety.

#### Delay discounting

Each participant was presented with a series of hypothetical scenarios of monetary gains where they indicated their preference for either a small, immediate reward or a larger, delayed reward. It should be noted that delay discounting research often employs hypothetical rewards, and that past research has found no significant differences in using hypothetical versus actual rewards (e.g., Madden, Begotka, Raiff, & Kastern, 2003; Madden et al., 2004).

The amount of the delayed reward remained fixed at $100 Canadian dollars (CAD) while the values of the immediate reward were $1, $2.50, $5, $7.50, $10, $15, $20, $25, $30, $25, $40, $45, $50, $55, $60, $65, $70, $75, $80, $85, $90, $92.50, $95, $97.50, and $99 CAD. The delay period for the delayed reward varied: ranging from 1 day, 1 week, 2 weeks, 1 month, 6 months, 1 year, and 5 years. For each delay period, participants were asked to indicate their preference between each immediate amount compared to the delayed amount by circling which reward they preferred. There were 175 trials in total (25 immediate reward amounts × 7 delay periods). Immediate amounts were presented in fixed descending order from the largest immediate reward to the smallest such that pairs of options were presented vertically down the page. Participants were presented with each delay in order of smallest delay to the largest, with each delay presented on a separate page. For each delay period, the lowest dollar value that a participant would take immediately, as opposed to waiting for the $100, was calculated as that participant’s switch point.

The decline in preference for the delayed rewards can be expressed by the following function developed by Mazur (1987):

$$ \mathrm{V}=\mathrm{A}/\left(1+\mathrm{kD}\right), $$

where the discounted value of the delayed reward (*V*), the delayed reward amount (*A*), and the delay in days (*D*) can all be used to estimate *k* which is equal to the slope of the delay curve and thereby describes how much value is affected by delay. Based on the above function, a curve was fitted to the 7 data points per participant (one for each time point), such that the slope parameter, *k*, was obtained. If *k* is relatively large, then the effect of delay (*D*) on degrading value is bigger than if *k* is small. A higher slope value (greater *k*) represents steeper discounting (a more rapid decrease in the value of a reward with increasing time, reflecting less delay of gratification) and this is used here as the dependent variable.^2^

### Results

Descriptive statistics for both measures can be found in Table 3. Twenty-two participants were excluded from the dataset due to incorrectly filling out the MMI (typically a failure to complete both sides of the diagonal in the matrix). An additional 34 participants were excluded for failing to meet the Johnson and Bickel (2008) inclusion criteria for delay discounting data (i.e., where responses did not allow the calculation of a single switch point for one or more delay periods given marked reversals of directions). For the remaining 150 participants, *k* values were estimated using MatLab scripts (Lau, 2011), and the resulting *k* values were log transformed to reduce markedly positive skew as is typical for *k* estimates (e.g., Alessi & Petry, 2003; Hariri et al., 2006)

**Table 3:** Descriptive data for Study-2 measures

Measure	*N*	*M*	*SD*	Minimum	Maximum
MMI score	150	4.89	1.93	.87	9.68
Delay discounting (log *k*)	150	−2.69	.86	−4.25	−.70

*MMI* Media Multitasking Index. Delay discounting mean represents log-transformed *k* (i.e., the degree to which each additional day decreases the perceived value of a $100 CAD reward

.

Zero-order correlations showed that higher MMI scores were associated with greater delay discounting (i.e., less delay of gratification), *r*(150) = .20, *p* < .05 as estimated using *k*. In addition to being positively associated with a greater discounting slope (*k*), MMI scores were negatively correlated with the switch point estimate at each of the seven delay intervals, indicating that those with higher MMI scores were willing to take lower amounts immediately instead of waiting, both at very short delay periods of a day or week through to very long delay periods of 1 or 5 years (all *p*s < .05).

**Fig. 2:**
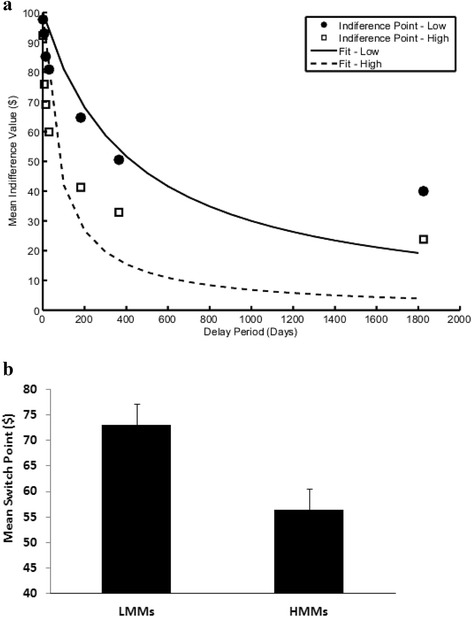
**a** Points represent the mean indifference point at each delay interval for heavy media multitaskers (HMMs) (high Media Multitasking Index (MMI) score group depicted with *open* symbols) and low media multitaskers (LMMs) (low MMI score group depicted with *filled* symbols). Lines represent the hyperbolic functions fitted to the data points for each group (*dashed* for HMMs and *solid* for LMMs). Note the lower indifference points and greater rate of decay for HMMs compared to LMMs indicate a greater willingness to settle for a smaller reward so as to get it immediately, reflecting a reduced ability to delay gratification. **b** Mean minimum number of dollars required to take the immediate reward (switch point) averaged across delay periods for LMMs and HMMs in Study 2. Error bars represent 1 standard error of the mean

As in Study 1, extreme groups were created based on MMI scores of greater or less than 1 SD from the mean (the 23 HMMs had MMI scores greater than 6.81, and the 20 LMMs had MMI scores lower than 2.97). An independent samples *t* test showed that HMMs (*M* = −2.37, *SD* = .82) had significantly higher discounting rates (*k* slopes) than LMMs (*M* = −2.90, *SD* = .86; *t*(41) = 2.03, *p* < .05, *d* = 0.62) reflecting HMMs’ preference for immediate gratification relative to LMMs’ (see Fig. 2a). Indeed, when mean switch points were averaged across all delay periods, LMMs required an average of $73.09 CAD now to forgo $100 CAD later, whereas HMMs required only $56.43 CAD now to forgo $100 CAD later, *t*(41) = 2.81, *p* < .05, *d* = 0.86 which reflects a meaningful, as well as a statistically significant difference in what the two groups were willing to take to get the reward immediately (see Fig. 2b).

## Discussion

In Study 1, HMMs were less likely to provide the correct answer to CRT questions and more likely to provide the automatic/intuitive answer than LMMs. This provides evidence that HMMs rely more on a fast and intuitive system-1 processing style that can be efficient, but also costly in terms of accuracy, rather than using the more deliberate and effortful system-2 processing. Greater media multitasking was also associated with greater self-reported impulsivity, supporting previous findings that HMMs are more impulsive (Minear et al., 2013; Sanbonmatsu et al., 2013). However, BIS scores (overall and each of the subscales) were unrelated to CRT scores; therefore, the relationship between MMI scores and CRT scores held even when controlling for BIS scores, suggesting that the CRT is not simply a behavioral measure of cognitive impulsivity.

A preference for more automatic and intuitive decision making, at the expense of more reasoned and effortful decision making, could have substantial real-world decision-making consequences, and has been shown to result in decisions favoring immediate gratification (Frederick, 2005). The results of Study 2 provide evidence that HMMs have a higher preference for immediate gratification than LMMs. Indeed, visual comparison of the delay discounting functions for HMMs and LMMs in Fig. 2 bears a striking similarity to previous findings showing reduced delay of gratification for those with various addictions (for example, see the discounting functions for problem gamblers and controls in Alessi & Petry, 2003). It appears that HMMs have a reduced ability for self-regulation in order to forego immediately gratifying rewards. This is consistent with the view that media multitasking is related to higher self-reported impulsivity and lower self-reported self-control (Minear et al., 2013; Sanbonmatsu et al., 2013).

### Why can’t they just put their phone down?

Previous studies have provided evidence that HMMs have increased self-reported mind-wandering and more everyday lapses of attention (Ralph et al., 2014), less top-down goal-related attentional selectivity (e.g., Ophir et al., 2009; Cain & Mitroff, 2011), greater self-reported impulsivity (Minear et al., 2013; Sanbonmatsu et al., 2013), reduced self-reported self-control (Minear et al., 2013), and, as observed here, an increased reliance on fast and automatic system-1 processing, and a reduced ability to delay gratification. We suggest that instead of proactively managing their approach to stimuli in the environment, HMMs may be more reactive – a pattern that has also been demonstrated in older-age participants (e.g., Braver & Barch, 2002; Schmitt, Ferdinand, & Kray, 2014), and which would be consistent with Loh and Kanai’s (2014) finding that HMMs have reduced gray-matter density in the anterior cingulate cortex relative to LMMs.

The direction of causality cannot be established here. It is possible that individuals who prefer immediate gratification are drawn towards media multitasking due to the its immediately gratifying nature. It is also possible that frequent media multitasking instead, or also, “trains the brain” to have a higher preference for immediate rewards due to habituation to the immediately gratifying nature of media multitasking.

Regardless of the reason for the association between media multitasking and delay discounting, the present results suggest that HMMs have a reactive decision-making style that promotes current desires (money, ease of processing) at the expense of accuracy and future rewards, providing empirical support for a hypothesis that has probably run through many of our minds on occasion when viewing others using media. Given that greater delay discounting is related to substance misuse, problematic gambling, overeating, and poor financial management (see MacKillop et al., 2011 for a review), the present results suggest that HMMs may be at risk for these behaviors as well, and it would be interesting to examine the frequency of these behaviors in LMMs versus HMMs. Furthermore, more investigation of the association between media multitasking and impulsivity, impaired delay of gratification, and system-1 thinking may help us understand how to curb dangerous multitasking trends with media, such as texting while driving. For example, if drivers who text while driving are less able to weight future consequences, such as harming themselves or others, compared to the immediate outcome of reading the text message, then this may suggest that media campaigns focused on future consequences may not be as effective for those most at risk for texting and driving. Even more benign media multitasking behaviors can have negative consequences, such as reduced academic or workplace performance, or social faux pas, and understanding the role of impaired delay of gratification and system-1 thinking in media multitasking may help us to understand the motivations behind these behaviors.

## Conclusions

Here, we observed that HMMs were more likely than LMMs to endorse intuitive, but wrong, decisions on the CRT indicating a greater reliance on quick and intuitive ‘system-1’ thinking and less reliance on slower and more effortful ‘system-2’ thinking. Heavy media multitaskers also displayed a reduced ability to delay gratification in a delay discounting task. Heavy media multitaskers were willing to take less money immediately relative to LMMs who were more willing to wait for the larger delayed reward. These results suggest that HMMs have a reactive decision-making style that promotes current desires (ease of processing, money) at the expense of accuracy and future rewards. These findings highlight the potential for HMMs to be at risk for problematic behaviors associated with immediate gains and longer-term negative consequences – behaviors such as substance abuse, overeating, problematic gambling, and impulsive spending – and further investigation of this association may suggest ways to remediate undesirable media multitasking behaviors.

## Abbreviations

BIS: Barratt Impulsivity Scale (BIS-11, Patton, Stanford, & Barratt, 1995)
CRT: Cognitive Reflection Test (a measure of system-1 thinking, see Frederick, 2005)
HMMs: Heavy media multitaskers (participants who score relatively high on the MMI)
LMMs: Light media multitaskers (participants who score relatively low on the MMI)
MMI: Media Multitasking Index (a measure of media multitasking behavior, see Ophir, Nass, & Wagner, 2009)

## Footnotes

1 Note that it is also possible, and indeed not infrequent, for participants to provide an answer that is incorrect, but not intuitive. Therefore, intuitive answers are not simply the difference between the total number of questions and the number of correct answers.

2
*k* is used here as it is the conventional dependent measure of choice in delay discounting studies. However, the same pattern of results was observed when delay discounting was estimated using an area under the curve measure (see Myerson, Green, & Warusawitharana, 2001) or when simply calculating the mean switch point across all delay intervals for each person. Indeed, once participants with inconsistent switch points were removed using the Johnson and Bickel (2008) criterion, these three measures were related to each other at *r* = .92 or better.
